# Picture Interpretation Test (PIT) 360°: An Innovative Measure of Executive Functions

**DOI:** 10.1038/s41598-017-16121-x

**Published:** 2017-11-22

**Authors:** Silvia Serino, Francesca Baglio, Federica Rossetto, Olivia Realdon, Pietro Cipresso, Thomas D. Parsons, Giacomo Cappellini, Fabrizia Mantovani, Gianluca De Leo, Raffaello Nemni, Giuseppe Riva

**Affiliations:** 1 Applied Technology for Neuro-Psychology Lab, IRCCS Istituto Auxologico Italiano, Via Magnasco, 2 20149 Milan, Italy; 2 Department of Psychology, Università Cattolica del Sacro Cuore, Largo Gemelli, 1, 20100 Milan, Italy; 3 IRCCS, Fondazione don Carlo Gnocchi ONLUS, Via Capecelatro 66, 20148 Milan, Italy; 4 Department of Human Sciences for Education, Università degli Studi di Milano-Bicocca, Milan, Italy; 5 Computational Neuropsychology and Simulation Laboratory, University of North Texas, 1155 Union Circle #311280, Denton, Texas 76203-5017 USA; 6 Department of Psychology, University of North Texas, 1155 Union Circle #311280, Denton, Texas 76203-5017 USA; 7 National Research Council of Italy, Institute for the Dynamics of Environmental Processes, Piazza della Scienza, 1, 20126 Milan, Italy; 8 Department of Clinical and Digital Health Sciences, College of Allied Health Sciences, Augusta University, 987 St. Sebastian Way, EC 4316, Augusta, Georgia 30912 USA; 9 Department of Pathophysiology and Transplantation, Università degli Studi di Milano, via Francesco Sforza, 35, 20122 Milan, Italy

## Abstract

The assessment of executive functions poses researchers with several challenges related to both the complexity of the construct of executive functions itself and/or the methodological difficulties related to its evaluation. The main objective of the current study was to evaluate a 360° version of an ecologically valid assessment called the Picture Interpretation Test (PIT). Participants included 19 patients with Parkinson’s disease (PD) and 19 healthy controls. All participants endorsed globally positive experiences of the PIT 360°. Furthermore, findings indicated that patients with PD took longer to correctly interpret the PIT 360° scene and tended to significantly focus on details of the 360° scene instead of the most informative elements. The time needed for a correct interpretation of the presented scene also correlated significantly with performance in conventional paper and pencil tests of executive functions for patients with PD. Classification analysis indicated the potential of the PIT 360° for distinguishing between patients with PD and healthy controls. Overall, these data provide preliminary evidence in support of the PIT 360° for evaluating executive functions.

## Introduction

The assessment of executive functions poses researchers with several challenges related to both the complexity of the construct of executive functions itself (see for example^1^) and/or the methodological difficulties related to its evaluation, specifically in predicting behaviors in real-life contexts^2–9^. In term of complexity, Chan and co-workers^10^ have described executive functions as “an umbrella term comprising a wide range of cognitive processes and behavioral competencies which include verbal reasoning, problem-solving, planning, sequencing, the ability to sustain attention, resistance to interference, utilization of feedback, multitasking, cognitive flexibility, and the ability to deal with novelty” (pg. 201). Others have proposed four cognitive constructs: volition, planning, purposive action, and effective performance^11^. Still others reduce this list to three cognitive constructs, namely inhibitory control, working memory, and cognitive flexibility^12–14^.

In an attempt to refine executive functions assessment, Burgess *et al*.^8^ advanced the idea of developing neuropsychological assessments based on models derived from directly observable everyday behaviors. Such an approach allows for an examination of the ways in which a sequence of actions leads to a given behavior in normal functioning. This “function-led” approach differs from the emphasis on abstract cognitive “constructs” without regard for their ability to predict the complexity of “functional” behaviors found in real-life situations^2–5^. Burgess and colleagues advanced the Multiple Errands test as a measure of executive functioning in real-life scenarios. While there are notable aspects of such naturalistic assessments, there are concerns about their many limitations in terms of time consumption, cost, poor control, and lack of safety^15^.

A potential alternative is the use of virtual reality (VR) technology for function-led assessments of executive functioning^16^. Indeed, VR permits the development of such assessments simulating everyday activities, allowing a secure and ecologically valid measure of executive functions^9,17^. A virtual Multiple Errands Test (VMET) has been developed and tested in various clinical populations^18–20^. The VMET allows for the evaluation of patients’ abilities in formulating and checking a list of goals to effectively respond to environmental demands to achieve a series of tasks (e.g., buy a specific product, ask the examiner information about a product to be purchased). A recent study found that VMET was an effective tool for detecting early executive deficits in non-demented patients with Parkinson’s Disease (PD)^19^. Furthermore, results demonstrated that patients with PD made more errors in the VMET tasks and showed a poorer ability in using effective strategies to complete the tasks in comparison to a control group. Interestingly, these two groups did not differ in their performance when compared on a traditional assessment of executive functioning.

A new technology for presenting neuropsychological stimuli is found in 360° environments (immersive photographs or videos) delivered via smartphones. The potentiality of 360° technology can be better understood by considering the “virtuality continuum” proposed by Milgram^21^, in which stimuli are presented in a manner ranging from completely real (real environment) to “virtual” (virtual environment). The space between extremes, called “mixed reality”, is the area wherein real and virtual may co-exist producing new experiences. Advances in 360° technologies allow participants to be immersed into a real situation that they experience from a first-person perspective. This platform allows for sequential focusing upon various elements and portions of the environment at different times. Moreover, this permits a sequential planning of visual search.

In this direction, we developed a 360° version of the Picture Interpretation Test (PIT)^22,23^ that leverages Luria and colleagues’^24^ use of a Russian picture entitled “Unexpected Return” to investigate active visual perception in patients with frontal lobe damage. The approach was developed from their belief that the interpretation of a meaningful picture engages the patient’s neurocognitive system via recursive selection of the most informative elements observed during the visual search. This allows for the elaboration and testing of hypotheses regarding its meaning. Rosci and colleagues^22^ validated the PIT for detecting executive deficits in an Italian sample of 196 normal adults and 12 patients with pre-frontal brain lesions, who were asked to interpret what was happening in a reproduction of the famous painting “Il Sorcio” (“The Mouse”). Findings revealed that 60 percent of the patients were unable to interpret the picture. Moreover, a similar failure rate was found in patient performance on a verbal fluency task and the Trail Making Test. These results suggest the potential of the Italian version of the PIT for testing of pre-frontal patients, thus making it one of the most used neuropsychological tests in the Italian context^25^.

The current study was aimed to evaluate a 360° version of the PIT for detecting executive deficits through a function-led approach that combines experimental control with real-world engaging background. The study included patients with Parkinson’s Disease (PD) because of the substantial research findings revealing a cognitive profile characterized by a dysexecutive syndrome^6,26,27^.

To investigate the quality of the experience associated with the experiencing the PIT 360°, we examined participant self-reports (e.g., perceived balance between challenge and skills, as well as patients’ intrinsic motivation in being confronted with the task). To specifically investigate the capability of PIT 360° for detecting executive deficits, we compared the performance of patients with PD to that of healthy controls (HC) using indices obtained from PIT 360°. Furthermore, we compared performance between the two groups on traditional construct-driven neuropsychological assessments of executive functioning. Finally, to evaluate the predictive validity of indices obtained from PIT 360°, we investigated how all these measures would be able to distinguish patients with PD from HC into their respective groups.

## Results

### User experience assessment

Table 1 presents descriptive data obtained from the user experience assessment divided between the two groups.

**Table 1:** Scores obtained from the user experience assessment for Parkinson’s Disease patient (PD Group) and Older Controls (HC Group). Data are shown as means and standard deviations (SD).

	PD Group	HC Group
GEW 1 *- Positive Valence/High coping potential* Number of emotion	2.368 (1.165)	2.842 (1.167)
GEW 1 *– Positive Valence/High coping potential* Intensity	3.303 (0.975)	3.575 (1.186)
GEW 2 *– Positive Valence/Low coping potential* Number of emotion	0.737 (0.872)	1.316 (1.455)
GEW 2 *– Positive Valence/Low coping potential* Intensity	1.210 (1.484)	1.570 (1.465)
GEW 3 – *Negative Valence/Low coping potential* Number of emotion	0.105 (0.315)	0.053 (0.230)
GEW 3 *– Negative Valence/Low coping potential* Intensity	0.210 (0.713)	0.053(0.230)
GEW 4 *– Negative Valence/High coping potential* Number of emotion	0.0	0.053 (0.230)
GEW 4 *– Negative Valence/High coping potential* Intensity	0.0	0.158 (0.688)
Perceived coping skills (Flow Short Scale)	3.368 (0.831)	3.158 (0.688)
Perceived challenge (Flow Short Scale)	2.684 (0.671)	2.474 (0.697)
Perceived challenge- skill balance (Flow Short Scale)	2.474 (0.612)	2.421 (0.692)
IMI	2.947 (0. 553)	3.189 (0.932)
SUS	4.1754 (1.496)	4.491 (1.517)

Comparison of data between groups using the Mann–Whitney U test did not reveal statistically significant differences (all *p*s > 0.05). Results obtained from the Friedman Test indicated a significant difference among the four quadrants of GEW in terms of the mean number of reported felt emotions [χ^2^(3) = 87.572; *p* < 0.001] and their intensities [χ^2^(3) = 91,377; *p* < 0.001]. Specifically, Wilcoxon tests on mean number of self-reported emotion within the different quadrants revealed that all participants experienced more emotions with positive valence and high goal conduciveness. The same findings resulted for the intensities of self-reported emotions. See Tables 2 and 3 for full statistics.

**Table 2:** Results obtained from Wilcoxon Test comparisons on different quadrants of Geneva Emotion Wheel. Mean number of reported felt emotion.

			U	p
*GEW 1*	2.065 (1.175)	vs. GEW 2	−4.944	<0.001
		vs. GEW 3	−5.218	<0.001
		vs. GEW 4	−5290	<0.001
*GEW 2*	1.026 (1.219)	vs. GEW 3	−3.082	<0.001
		vs. GEW 4	−3.872	<0.001
*GEW 3*	0.079 (0273)	vs. GEW 4	−1.000	0.317
*GEW 4*	0.026 (1.622)

**Table 3:** Results obtained from Wilcoxon Test comparisons on different quadrants of Geneva Emotion Wheel. Intensities of reported felt emotion.

			U	p
*GEW 1*	3.439 (1.080)	vs. GEW 2	−5.105	<0.001
		vs. GEW 3	−5.176	<0.001
		vs. GEW 4	−5.249	<0.001
*GEW 2*	1.390 (1.466)	vs. GEW 3	−3.561	<0.001
		vs. GEW 4	−3.939	<0.001
*GEW 3*	0.131 (0.528)	vs. GEW 4	−5.577	0.577
*GEW 4*	0.080 (0.487)			

### Conventional neuropsychological assessment

Table 4 offers an overview of the scores obtained from the traditional neuropsychological evaluation divided between HC and patients with PD.

**Table 4:** Scores obtained from the neuropsychological assessment for Parkinson’s Disease patient (PD Group) and Older Controls (HC). Data are shown as means and standard deviations (SD).

	PD Group	HC
Montreal Cognitive Assessment (MoCa)	26.000 (1.732)	27.789 (2.149)
Trail Making Test (TMT-A)	51.316 (24.637)	35.684 (17.327)
Trail Making Test (TMT-B)	135.824 (108.449)	80.263 (43.252)
F.A.S. Verbal Fluency	34.158 (9.800)	40.684 (9.939)

When controlling for age and education, results showed that the PD group had significantly lower scores on MoCa when compared to HC [F(1, 34) = 10.252; p = 0.003; Partial η^2^ = 0.232], they also performed significantly poorer on the phonemic fluency task [F(1, 34) = 6.390; p = 0.016; Partial η^2^ = 0.158) and in the two TMT sub-tests, namely the TMT-A [F(1, 34) = 7.075; p = 0.012; Partial η^2^ = 0.172] and the TMT-B [F = (1, 34) 4.240; p = 0.047; Partial η^2^ = 0.111)].

### Performance on PIT 360°

Fourteen patients with PD (73.7%) correctly interpreted the scene proposed in the PIT 360°, while 5 patients with PD (26.3%) failed in the recognition. As concerns the HC group, sixteen participants (84.2%) recognized the scene, while 3 participants (15.8%) didn’t succeed in the task. There was no significant difference in the proportion of participants successfully completing the task between groups [χ^2^(1) = 0.426; *p* = 0. 693].

When controlling for age and education, results showed that patients took longer (mean = 106.418; SD = 66.851) in comparison with HC group (mean = 70.808; SD = 52.782) for giving an interpretation of the scene proposed [F(1,34) = 4.624; *p* = 0.039; Partial η^2^ = 0.120]. Moreover, the PD group (mean = 10.00, SD = 7.039) provided a more detailed description of the scene in comparison to the HC group (mean = 5.789; SD = 3.505) [F(1,34) = 5.695; *p* = 0.023; Partial η^2^ = 0.143].

### Correlations between neuropsychological tests and performance on PIT 360°

**Figure 1:**
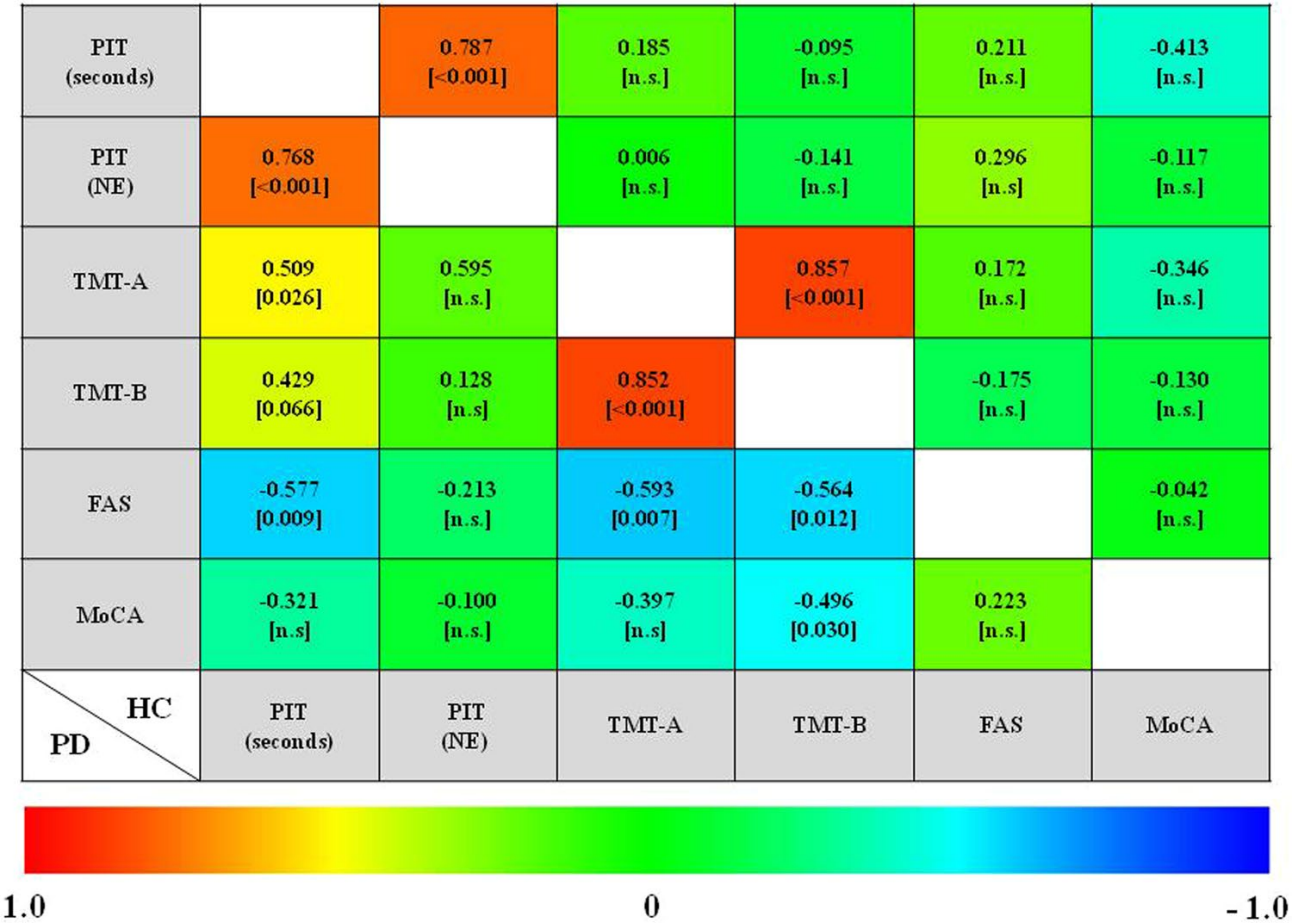
Correlation between neuropsychological tests and performance on PIT 360°. Correlation coefficients are represented by a color, which ranges from red (1) to blue (−1). Correlations between neuropsychological tests and indexes of PIT 360° for healthy controls (HC) are shown in the upper right, while findings for patients with Parkinson’s Disease (PD) are displayed in the bottom left.

There were no significant correlations between neuropsychological tests and indexes of PIT 360° for HC group (see Fig. 1). For patients with PD, the time needed for a correct interpretation of the PIT 360° (Correct Interpretation) was found to positively correlate with performance on TMT-A (r = 0.509; *p* = 0.026) and negatively with that on the phonemic fluency task (r = −0.577; *p* = 0.009) (see Fig. 1). At the same time, there was a trend for correlation between Correct Interpretation and TMT-B (r = 0.429; *p* = 0.066).

### Classification of Healthy Controls or Patients with PD

Performance of the classifiers was evaluated by carrying out a relative operating characteristic (ROC) analysis^28^. The area under the ROC curve (AUC) provides a single measure of overall prediction accuracy, *Precision* represents the proportion of true positives among all the instances classified as positive, *CA* (Classification accuracy) represents the proportion of the instances that were classified correctly, *F1* indicates the harmonic mean of precision (P) and Recall (R), and *Recall* (R) is the proportion of cases which were classified as positive, among all instances which truly were positives. Results from nonlinear stochastic approximation (i.e., machine learning approach) methods showed a Precision between 55.6% and 68.8% for the conventional neuropsychological assessment of executive functions (Table 5), while it ranged from 50.0% to 71.4% for PIT 360° (Table 6). According to these results (Tables 5 and 6), Random Forest has a Precision under the 60%, thus making this algorithm not reliable for the classification of cases into two groups using both traditional neuropsychological tests and indices from PIT 360°. On the other hand, results obtained with the Logistic Regression showed a good Precision only for traditional neuropsychological tests, but not for PIT 360°. Finally, we opted to use both Support Vector Machine and Naïve Bayes that showed a good precision (over than 60%).

**Table 5:** Stratified 10-fold Cross validation for the neuropsychological assessment battery^1^.

Method	AUC	CA	F1	Precision	Recall
Logistic Regression	0.658	0.658	0.629	0.668	0.579
Random Forest	0.533	0.533	0.541	0.556	0.526
Support Vector Machine (SVM)	0.658	0.658	0.667	0.650	0.684
Naïve Bayes	0.632	0.632	0.632	0.632	0.632

^1^AUC (Area under the ROC curve) is the area under the classic receiver-operating curve; CA (Classification accuracy) represents the proportion of the examples that were classified correctly; F1 represents the weighted harmonic average of the precision and recall (defined below); Precision represents a proportion of true positives among all the instances classified as positive. In our case, the proportion of condition correctly identified; Recall represents the proportion of true positives among the positive instances in our data.

**Table 6:** Stratified 10-fold Cross validation for the indices of PIT 360°^1^.

Method	AUC	CA	F1	Precision	Recall
Logistic Regression	0.579	0.579	0.556	0.588	0.526
Random Forest	0.500	0.500	0.457	0.500	0.421
Support Vector Machine (SVM)	0.605	0.605	0.516	0.617	0.421
Naïve Bayes	0.658	0.658	0.606	0.714	0.526

^1^AUC (Area under the ROC curve) is the area under the classic receiver-operating curve; CA (Classification accuracy) represents the proportion of the examples that were classified correctly; F1 represents the weighted harmonic average of the precision and recall (defined below); Precision represents a proportion of true positives among all the instances classified as positive. In our case, the proportion of condition correctly identified; Recall represents the proportion of true positives among the positive instances in our data.

**Figure 2:**
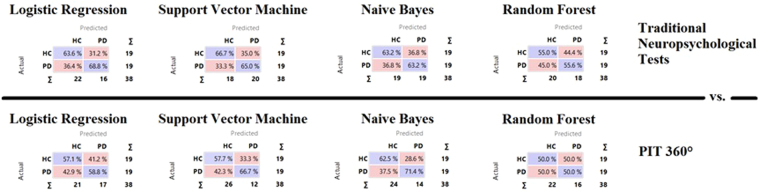
Classification of Healthy Controls or Patients with PD. The diagonal values (i.e., purple values) represent the elements for which the predicted group is equal to the true group, while off-diagonal elements are those that are mislabeled by the classifier. Naïve Bayes and Support Vector Machine algorithms demonstrated that PIT 360° has a higher capability in predicting PD Group membership with respect to traditional neuropsychological tests of executive functioning

Although the ability to predict control group membership is quite similar between the two types of assessment (slightly better for the traditional neuropsychological assessment), it is interesting to note that Naïve Bayes and Support Vector Machine algorithms showed that the indices from PIT 360° had a higher capability in predicting PD Group membership (see Fig. 2).

## Discussion

The main objective of the current study was to evaluate the 360° version of the Picture Interpretation Test (PIT)^22,23^, for providing assessment of executive functions processing in PD using an innovative and ecologically valid tool. Following immersion in the PIT 360°, HCs and patients with Parkinson’s Disease (PD) were surveyed on their affective reactions to the 360° scene, perceived level of challenge and skills, appreciation of the tool, and their sense of presence while immersed in the PIT 360°. Then, to specifically evaluate the ability of PIT 360° in detecting executive deficits, we compared the performance of patients with PD and healthy controls comparing conventional neuropsychological assessments with the PIT 360°. Correlations between the conventional neuropsychological tests of executive functions and performance on PIT 360 were also explored. Finally, we investigated the predictive validity of indices obtained from PIT 360° in distinguishing PD patients from the healthy controls. Results from user experience assessment of the PIT 360° revealed that all participants endorsed positive reactions to their experience of the PIT 360°. This was apparent in the higher scores in the first quadrants of Geneva Emotion Wheel (GEW^29,30^), which includes interest, joy, happiness, satisfaction, elation and pride. In particular, patients with PD did not endorse affective responding with low valence and high control (such as anger or irritation). Moreover, both groups perceived a high level of their own skills in the context of a demanding task (the interaction with PIT 360°), which resulted in perceived balanced level of challenge-skills. As emerged by mean scores of Intrinsic Motivation Inventory (IMI^31^), the PIT 360° was reported to be an interesting and an enjoyable activity. Finally, all participants reported a very high level of presence during the interaction with PIT 360°.

All subjects were preliminarily assessed by a neuropsychological assessment and all of them obtained scores within the normal range. This confirms that our patients were in a relatively well-preserved clinical state. Only a statistical group comparison revealed differences between the two groups. As expected, these behavioral findings indicate that patients with early PD and no clinical evidence of cognitive impairment may already exhibit sub-clinical abnormalities, as previously reported^32,33^. In line with the pattern of results from conventional neuropsychological assessment, the PIT 360° analysis revealed different performances in patients with PD compared to HC. Although the percentage of PD patients that failed in correctly interpreting the scene is quite similar to that of HC (26.3% *vs*. 15.8%) confirming that they showed a relatively well-functioning cognitive status, analyses on the two PIT 360° indices showed significant differences between the two groups. Specifically, patients with PD took longer to provide a correct interpretation of the scene proposed and provided significantly more details about the objects found in the scene. While the patients gave significantly richer descriptions of the scene, they appeared more prone to distractor interference (“There is a white coat, there are two chairs in front of the TV. There is a big TV. On the floor, there is maybe a scale. Then, I see a wardrobe that may be a fridge. There is a man who is working on jumper cables near the wardrobe. I see an electric device on the table. I think that the man is repairing something. The man is curled up behind a white-board, or something similar, a spot where it is possible to hang sheets.”). These findings are in line with Luria’s view^24^, suggesting that this test is able to capture deficits in active visual perception. Our data indicated that patients with PD demonstrated more difficulties when compared to healthy controls in focusing on the most important components for a correct interpretation of the scene. Thus, PD patients appear to focus on details instead on the most informative elements. They were not able to find important elements for a correct interpretation of the whole scene nor did they match these elements with their hypotheses about the meaning. In most cases, a poor interpretation based only on the details was given.

Interestingly, results from correlation analyses indicated that neuropsychological tests correlate significantly with indexes of PIT 360° only for patients with PD. Specifically, the time needed for giving a correct interpretation of the PIT 360° scene did not correlate with the Montreal Cognitive Assessment (i.e., a measure of global cognitive level), but it was significantly correlated with the Trail Making Test and the phonemic verbal fluency task, thus tapping both verbal and visuospatial aspects of executive functioning and motor aspects. These findings suggest that PIT 360° can be considered as a quick, ecological and useful screening instrument able to evaluate different aspects of dysexecutive impairment in patients with PD.

Results obtained from classifiers clearly indicated the potential of PIT 360° scene assessment in distinguishing between patients with PD and HC. Two of the algorithms used indicated that PIT 360° had a higher capability in predicting PD group membership with respect to a traditional neuropsychological assessment. Although machine learning approaches have been traditionally applied to the analysis of very complex medical datasets^34^, recent studies have also applied them for classifying patients according to their cognitive impairment and consequently reduce the number of onerous tests required for their diagnosis^35–37^.

While the findings of the current study are promising, there are some limitations that should be considered. First of all, in order to fully evaluate the potentiality of PIT 360° as a new screening tool of executive functions, future studies are needed to assess its test–retest reliability and validity. A large validation study with a sample of participants across the lifespan including the PIT 360°, the original PIT^22,23^, as well as other conventional neuropsychological measures should be performed. Moreover, it will be important to investigate the value of PIT 360° in detecting executive impairments in other clinical populations who are known to have executive dysfunctioning.

## Conclusions

This study provides the first evidence that the 360° technology may play a role in the future of neuropsychological assessment. Moreover, this technology may be integrated with other portable devices, such as an eye-tracker. As suggested by pioneering study of Luria^24^, it would be particularly interesting to investigate patients’ eye movements during the interpretation of the scene proposed. Indeed, Luria found that disturbances in the active visual perception were reflected by a corresponding disorganized scanning gaze movements. In conclusion, although preliminary, our findings provide encouraging evidence in support of the use of immersive 360° environments in general, and the PIT 360° specifically for innovative evaluation of executive impairments.

## Materials and Methods

### Participants

Thirty-eight participants took part in the study: 19 patients with Parkinson’s Disease (PD group), and 19 healthy controls matched for age and education with the PD group (HC group).

Outpatients meeting the diagnostic criteria for probable PD^38^ were consecutively recruited from the Neurorehabilitation Unit of Don Carlo Gnocchi Foundation, IRCCS. All patients were at a mild to moderate stage of the disease, scoring between stages 1 and 2 of the Hoehn and Yahr (H&Y) Scale^39^. None had any report of cognitive problems or any evidence of cognitive deficits in their daily living activities. A Mini Mental State Examination (MMSE) was used to exclude any patient who reported scores outside the normal range (MMSE cut-off score 23, 8^40,41^). All subjects (patients and HCs) were right handed as assessed by the Edinburgh Inventory^42^. Exclusion criteria included any major systemic, psychiatric, or other neurological illnesses. Particular attention was used to exclude those patients who experienced visual hallucinations, had episodes of severe depression or autonomic failure, manifested resistance to dopaminergic drugs and were at an unstable dosage of antiparkinsonian treatment during the 3 months prior to study entry. The PD group was composed of 3 women and 16 men, while the HC group included 9 women and 10 men. The mean age for the PD group was 66.53 (SD = 9.43), with an average of 12.47 (SD = 3.47) years of education of, while the mean age for the HC group was 67.58 (SD = 7.86), with an average of 14.37 (SD = 3.48) years of education. The two groups did not differ significantly in terms of age [t(36) = −0.377; *p* = 0.708] or education [t(36) = −1.680; *p* = 0.102]. However, there were significantly less women in the PD group [χ^2^(1) = 4.835; *p* = 0.036].

The study was conducted in compliance with the Helsinki Declaration of 1975, as revised in 2008. Local Ethics Committee (Don Carlo Gnocchi Foundation) approval and written informed consent to be included in the study was obtained by participants before study initiation.

### Procedure of the study

Participants underwent a conventional neuropsychological assessment to obtain their global cognitive profile and level of executive functioning (pre-task evaluation). Subsequently participants were asked to complete the PIT) 360° (PIT 360° session). The PIT 360° was designed and administered through an innovative mobile application (PIT 360) that allows participants to explore an immersive 360° experience. At the end of PIT 360 task evaluation, subjects were asked to rate their affective reactions, perceived levels of challenge and skill, appreciation and sense of presence experienced while performing the PIT 360 task (post-task evaluation).

#### Pre-task evaluation: neuropsychological measures

In the pre-task evaluation we administered the following conventional paper and pencil tests: the Montreal Cognitive Assessment (MoCa^43^) as a measure of global cognitive level; the Trail Making Test (in two specific sub-tests: TMT-A and TMT-B^44^) and the phonemic verbal fluency task (FAS^45^) as measures of executive functioning.

#### PIT 360° session: The PIT 360° development, description and administration

The PIT 360° is the 360° version of the Picture Interpretation Test^22,23^. In the PIT test, a small-scale color reproduction (19 × 13) of the famous painting “Il Sorcio” (“The Mouse”) of the Italian painter Giacomo Favretto is used as a test stimulus. In this painting, a room in disarray is presented with three frightened girls standing on chairs and a boy who is searching for something on the floor. Although not visible, it is apparent that there is mouse hidden behind a piece of furniture. Participants are asked to interpret what is happening in the scene in a limited time frame (180 seconds), while the time to say the word “mouse” is the outcome measure.

**Figure 3:**
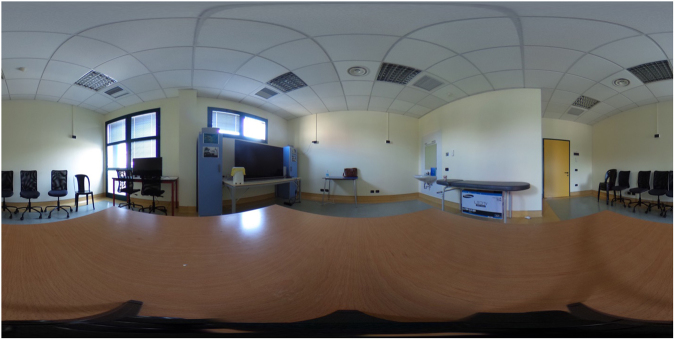
PIT 360°. Familiarization phase.

**Figure 4:**
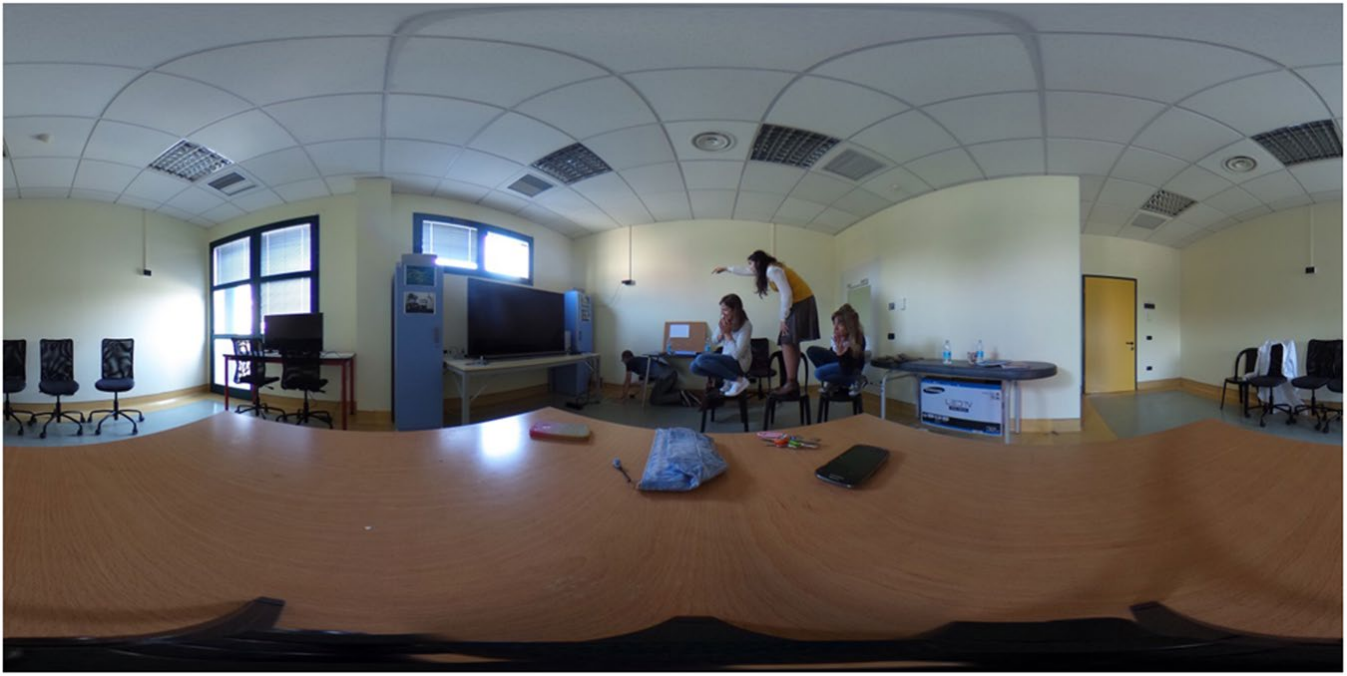
PIT 360°. Testing phase.

The PIT 360° was developed with the Ricoh Theta S Digital Camera that permits the creation of 360° spherical imageries. The camera is able to capture a 360° scene by stitching two 180° scans via integrated software at a resolution of 1792 by 3584 pixels. This allows for a presentation of an immersive stereoscopic 360° experience directly on a virtual reality headset (including mobile phone) via the Ricoh Theta S application. For the present study, the PIT 360° was rendered trough the mobile application of the Ricoh Theta S on an iPhone 6 Plus. Two scenes were recorded: one, to be used in the Familiarization phase (see Fig. 3), in which a meeting room appeared with tables, chairs, a sink, a television, some dressers with several objects spread on them. The second scene (see Fig. 4), to be used in experimental phase, was designed in line with the Favretto’s painting “Il Sorcio”: in the same room, with the same furniture and objects spread throughout it along with a boy searching for something on the floor, while three frightened girls standing on chairs watch him.

The neuropsychologist started the administrations by inviting participants to sit on a swivel chair and to wear the virtual reality headset (connected to the iPhone 6 Plus). This allowed participants to explore an interactive 360° experience. In case of presbyopia, participants were asked to wear their own glasses. Then participants underwent a familiarization phase (3 minutes) aimed at familiarizing them with the technology and control for potential side effects (e.g., dizziness, nausea). The examiner followed a cessation rule in which experimental sessions should be stopped if severe side effects occurred. The examiner asked participants to keep their eyes closed, and started time registration (in seconds) and audio recording coinciding with the instruction “Open your eyes”. Participants were then presented with the 360° scene of the room including a table (in the center), a sink with a mirror (on the participant’s right), a television on a table, two dressers (on the participant’s left), and various chairs and objects spread throughout the room. They were asked to find five objects in the scene to answer the experimenters’ questions (i.e. “Let’s search for the agenda. Where is the agenda?”). Upon completion of the three-minute familiarization phase, participants were asked to close again their eyes. The experimental session began with time registration (in seconds) and audio recording coinciding with the examiner’s instruction “Open your eyes”. In this phase, participants were asked to freely explore the scene derived from Favretto’s “Il Sorcio” and to tell the examiner what is happening as quickly as possible (maximum time: 180 seconds). Time registration lasted until the instant in which the participant said the word “mouse” or something similar (e.g., “snake”, “roach”, etc; generic classifications were allowed). After participants pronounced the word “mouse” (or similar generic classifications), the experimenter asked “What do you mean?” in order to confirm the participant’s understanding of the situation.

The following indices were calculated:

1. *Correct Interpretation*: The time in seconds registered from the time in which the experimenter said the words “Open your eyes” until the participant provided a correct interpretation” (i.e., “mouse”, “animal”, etc.). The maximum time allowed was 180 seconds. If the participants failed to interpret the scene in the allotted 180 seconds, then a time of 180 seconds was assigned as the outcome (as suggested by Rosci and colleagues^22^);
2. *Number of Scene Elements*: The sum of the scene elements that were verbalized during the interpretation of the scene.

#### The post-task evaluation: user experience assessment

In the post-task evaluation, participants were asked to rate their experience during the task on the following instruments:

Geneva Emotion Wheel (GEW^29,30^).

It consists of 20 discrete emotion terms that are systematically aligned in a circle. Underlying the alignment of the emotion terms are the two dimensions – valence/goal conduciveness (negative to positive) and control/coping potential (low to high) separating the emotions into four quadrants, each meant as an emotion family: Positive valence/High coping potential, Positive valence/Low coping potential, Negative valence/Low coping potential, and Negative valence/High coping potential. In all the four quadrants, the single emotion terms are considered as indexes “reflecting a unique experience of mental and bodily changes in the context of being confronted with a particular event”^29^. The mean number of emotions labels chosen within each of the four quadrants and the reported intensity in feeling it show how participants shaped their subjective feeling in performing the PIT 360° task along the dimensions of valence/goal conduciveness and control/coping potential.

Perceived fit of demands and skills (from Flow Short Scale^46^). Landhäußer and Keller^47^ highlighted that, although researchers seem to equalize the skill-demands compatibility with the experience of flow itself (e.g.^48,49^) in many studies, the balance between skills of the individual and perceived challenges of the task cannot be considered as a measure of the flow experience per se. Therefore, we administered the three items from the Flow Short Scale (5-points scale) that assess this specific component of the flow experience in performing the PIT 360° task. The first item asked participants to evaluate their perceived level of skills in coping with the task (“Perceived coping skills”), whereas the second item is related to the perceived level of challenges (“Perceived challenge”). Finally, participants were asked to indicate the perceived challenge-skill balance (“Perceived challenge- skill balance”) in 5-point-scale with 1 indicating that the current challenge is too low for ones’ perceived skills, 3 indicating that the current challenge fit exactly to ones’ skills and 5 indicating that the challenge is too high.

Intrinsic Motivation Inventory (IMI^31^). Participants responded to five items (7-points scale) from the subscale “Enjoyment” of the Intrinsic Motivation Inventory (IMI). These items were chosen to evaluate participants’ appreciation to the proposed activity, including the items “This activity was fun to do” and “While I was doing this activity, I was thinking about how much I enjoyed it”. The mean of the item scores is considered.

The Slater-Usoh-Steed Questionnaire (SUS^50^). It consists of 7-points questionnaire which evaluates the sense of presence with three items: 1) the sense of being in the scene depicted in the 360° scene, 2) the extent to which the 360° scene became the dominant reality, and 3) the extent to which the 360° scene was remembered as a place. The mean of the item scores was considered.

### Data analysis

The normality of data distribution was assessed using the Kolmogorov-Smirnov test. Since data were not normally distributed, non-parametric tests were used to investigate the quality of the experience associated with the interaction with PIT 360° and potential differences between PD and HC group on user experience variables (i.e., GEW, Flow Short Scale, IMI, and SUS). Moreover, for the GEW^29,30^, differences within the four quadrants in the number and intensity of self-reported emotions were also investigated using the Friedman Test. Next, Wilcoxon tests, with Bonferroni’s adjustment, were computed to break down significant findings. Subsequently, between-group comparisons of performance on the conventional neuropsychological assessment and the indexes of PIT 360° (Correct Interpretation and Number of Scene Element) were made by univariate analysis of covariance (ANCOVA), using age and education as covariates. These statistical analyses were performed using the Statistical Package for the Social Sciences for Windows (IBM Corp Armonk, NY, USA), version 23. Finally, Pearson correlation coefficient was used to examine correlations between conventional neuropsychological tests and the indexes of PIT 360°. These statistical analyses were carried out using the software MedCalc (MedCalc Software, Ostend, Belgium), version 16.8.4.

Nonlinear stochastic approximation (i.e., machine learning) methods were used to compare the classification accuracy of traditional neuropsychological assessments versus the PIT 360° indices for classifying participants into either the “Patients with PD” or “Healthy Controls” groups. Machine learning approaches are devoted to prediction and it is thought to be explorative rather than explicative^51^; accordingly, we used different algorithms to compare the predictive value of each one of them to understand which one was the best based on their accuracy. Because our analyses are based on relatively small sample sizes, a Leave-one-out cross-validation (LOOCV)^52,53^. Different algorithms were employed, namely: a) a Logistic Regression classification algorithm with ridge regularization; b) a Random Forest classification to classify using an ensemble of decision trees; c) a Support Vector Machine (SVM) to map inputs to higher-dimensional feature spaces that best separate different classes; d) a Naïve Bayes classification, for discriminating between the two groups, even without any particular assumption for the distribution for the features. All these analyses were computed using Python 3.4 with the Orange 3.3.5 data mining suite, which is freely available as open source code (https://github.com/biolab/orange3).
